# From Grave to Cradle: Kombucha Waste for Sustainable Electronics

**DOI:** 10.1002/advs.202514521

**Published:** 2025-10-17

**Authors:** Xin Ying Chan, Xiaolu Sun, Eddy Yi Ler Pang, Iris Zhiyu Ren, Xuan Zhang, Pengyu Chen, Yu Jun Tan

**Affiliations:** ^1^ Department of Mechanical Engineering College of Design and Engineering National University of Singapore Singapore 117575 Singapore

**Keywords:** bacterial cellulose, biodegradable wearable, eco‐friendly fabrication, kombucha, sustainable electronics

## Abstract

The increasing reliance on petroleum‐based polymers in electronics contributes significantly to electronic waste. Kombucha bacterial cellulose (KBC), a renewable and compostable byproduct of kombucha fermentation, presents an eco‐friendly alternative. However, the absence of sustainable fabrication methods for high‐performance KBC films has limited their electronic applications. This study introduces an environmentally benign process for pulping, purifying, and forming KBC sheets optimized for sustainable electronics. Treatment with sodium bicarbonate and hydrogen peroxide yields a sterile, white KBC film with enhanced properties. Comprehensive characterization via TGA, XRD, and FTIR confirms the material's high purity and crystallinity while preserving its native chemical structure. Mechanical testing demonstrates that processed KBC films exhibit superior tensile strength compared to untreated samples. Additionally, a gold‐sputtering technique is established to create conductive circuits on KBC substrates, achieving stable electrical conductivity even under mechanical stress. As a proof of concept, this platform, in a functional pressure sensor for flatfoot assessment, is successfully implemented. A key advantage of KBC is its rapid biodegradation, completing the material lifecycle within days. These results position KBC as a promising, sustainable biomaterial for next‐generation green electronics.

## Graphical abstract

This study introduces a sustainable method to purify kombucha bacterial cellulose (KBC) with sodium bicarbonate and hydrogen peroxide, avoiding harsh chemicals. KBC films are patterned with gold and function as pressure sensors for flatfoot gait detection. At the end‐of‐life, the KBC‐based device biodegrades in soil, leaving environmentally benign gold residues, while other electrical components are recovered for reuse, ensuring sustainability.

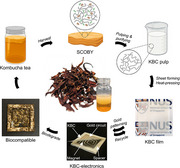

## 1 Introduction

Humanity's growing reliance on modern electronics has led to a troubling array of environmental challenges, including the massive generation of electronic waste, largely driven by the increasingly rapid turnover of devices as consumers upgrade to newer models every few years.^[^ ^1^ ^]^ At the heart of the problem lies the widespread use of petroleum‐based polymers, which are indispensable components in electronics, functioning as insulation or passivation materials for preventing signal interference, reducing delays in resistance‐capacitance circuits, and dissipating energy.^[^ ^2^ ^]^ Unfortunately, these petroleum‐based polymers are derived from non‐renewable sources and require energy‐intensive processing before being incorporated into electronic devices. At the end of their lifecycle, they often accumulate in landfills, as they are neither biodegradable nor recyclable.^[^ ^3^ ^]^ Furthermore, toxic components can leak into the nearby ecosystem, posing serious threats to both wildlife and human health.^[^ ^3^, ^4^ ^]^ Therefore, there is an urgent need to find alternative sustainable materials to replace petroleum‐based polymers used in electronics.

Kombucha bacterial cellulose (KBC) offers a promising paradigm as a biopolymer produced as a waste byproduct of the kombucha tea fermentation process, which is often discarded.^[^ ^5^, ^6^ ^]^ Repurposing this surplus, KBC not only offers a more economical and sustainable cellulose source but also addresses concerns over waste generation. KBC has previously been reported in use as packaging materials,^[^ ^7^ ^]^ wound dressings,^[^ ^8^ ^]^ textile materials,^[^ ^9^ ^]^ and electronics.^[^ ^10^, ^11^ ^]^ Despite existing research, key concerns regarding the long‐term property changes of KBC remain largely unaddressed. This issue is particularly pronounced in wet and unpurified KBC. Furthermore, since KBC is derived from a natural film‐forming process, it often exhibits slight variations in physical properties such as thickness and mass, making it difficult to be applied in applications requiring high consistency. Even when purified, conventional treatment usually involves sodium hydroxide (NaOH)‐based and sodium hypochlorite‐based bleaching,^[^ ^12^ ^]^ which involves harsh and potentially hazardous chemicals that are not environmentally friendly.^[^ ^13^ ^]^ Therefore, ensuring a milder purification treatment for KBC is crucial for achieving consistent and reliable performance, enabling its use across a wide range of applications as a sustainable alternative.

Herein, we demonstrated an eco‐friendly method of producing KBC with consistent properties through a mild purification treatment using sodium bicarbonate (baking soda, BS) and hydrogen peroxide (H_2_O_2_). Our KBC achieved whiteness, chemical and mechanical properties comparable to those produced through conventional bleaching, but with significantly shorter processing time, no need for heating, and minimal water usage. We further demonstrated KBC as a substrate in biodegradable flexible electronics and developed a technique to pattern conductive materials on it. KBC was also applied as a pressure sensor for monitoring flatfoot conditions. The KBC devices are biocompatible and biodegrade safely to environmentally benign components at their end‐of‐life. This study represents the first efforts to repurpose and evaluate the performance of KBC throughout its entire lifecycle as a sustainable biopolymer, offering an alternative to petroleum‐based materials for sustainable electronic applications derived from waste materials.

## 2 Results and Discussion

**Figure 1:**
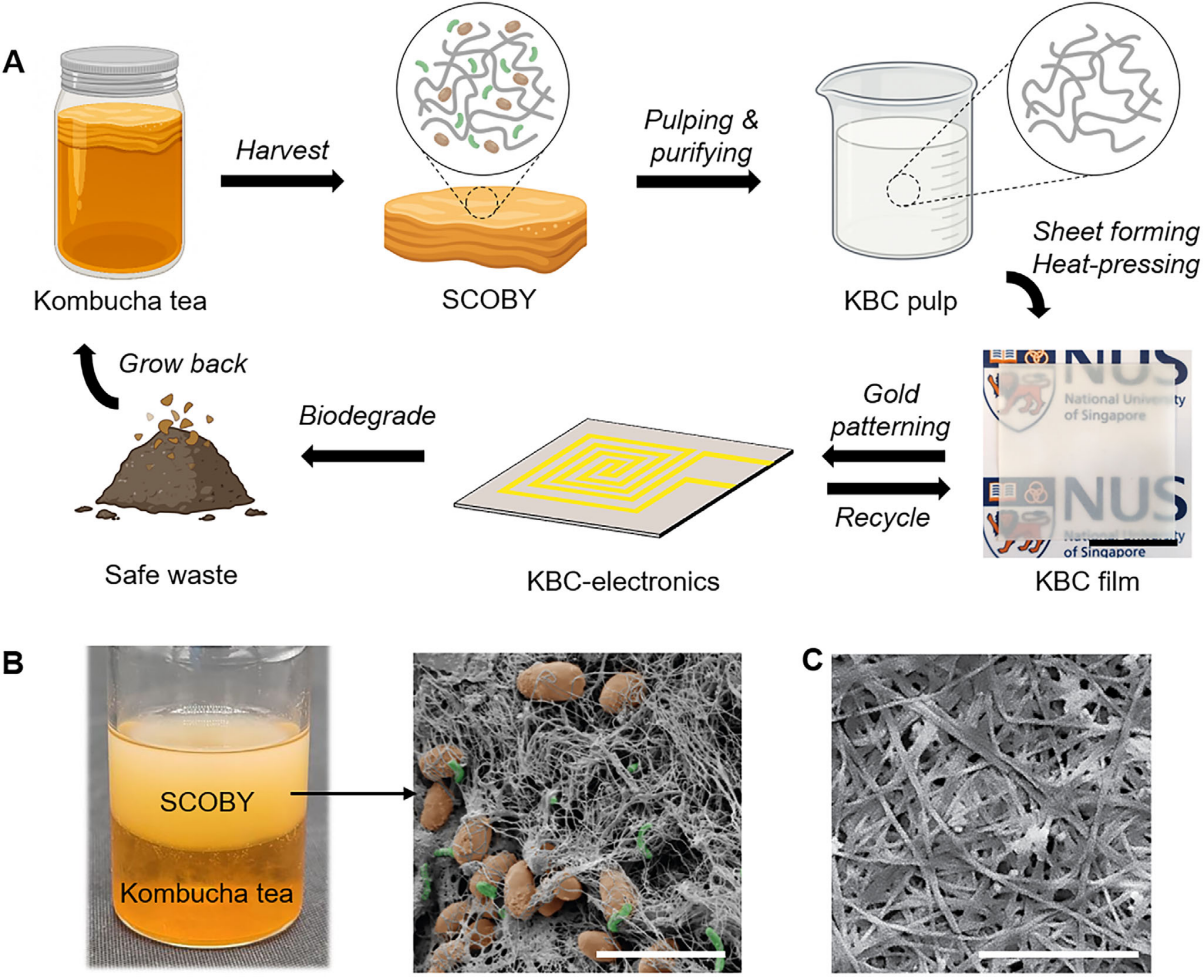
Lifecycle and morphology of KBC and KBC‐based electronics. A) Schematic illustrating the lifecycle of KBC and KBC‐electronic devices. The SCOBY pellicle is harvested from kombucha tea fermentation, then purified and reconstructed into a thin, white film, which serves as a sustainable substrate for mounting electronic components. After use, the KBC substrate can be either recycled or biodegraded under standard composting conditions, minimizing environmental impact. Scale bar: 5 cm. B) Photograph showing SCOBY formation at the tea‐air interface. False‐colored FESEM image depicts the morphology of the unpurified SCOBY pellicle, with yeast and bacteria highlighted in brown and green, respectively. Scale bar: 10 µm. C) FESEM image of a purified KBC film, revealing a clean cellulose nanofibrous network free of microbial residues. Scale bar: 1 µm.

The life cycle of KBC is illustrated in **Figure** **1**A, beginning in the “grave” as a waste byproduct of kombucha tea fermentation. During this process, a symbiotic culture of bacteria and yeast (SCOBY) forms a thick pellicle at the air‐liquid interface of each batch (Figure 1B; Figure S1, Supporting Information). This biologically active pellicle contains living microorganisms and continues to undergo fermentation, making it unsuitable in its raw form for electronic applications.

To convert the SCOBY into a material compatible with electronics, a purification and reconstruction process was developed to produce a uniform, stable, and biodegradable KBC film. Field emission scanning electron microscopy (FESEM) of the unprocessed SCOBY reveals a porous cellulose network with nanofibers ≈70 nm in diameter, along with embedded yeast and bacteria (Figure 1B). In contrast, after purification, the resulting KBC film displays a clean and densely packed cellulose nanofiber matrix, free of microbial or organic impurities (Figure 1C). This densification is attributed to the heat‐pressing step during film reconstruction.

We optimized the yield of SCOBY pellicles by adjusting the composition of the fermentation medium. The highest yield, 248 g L^−1^, was achieved using 30% kombucha tea with 15% added sugar (Figure S2, Supporting Information). For purification, we developed a mild and environmentally friendly method using BS followed by H_2_O_2_, avoiding the conventional use of NaOH. This method effectively sterilized, whitened, and removed unstable compounds from the pellicle.

**Figure 2:**
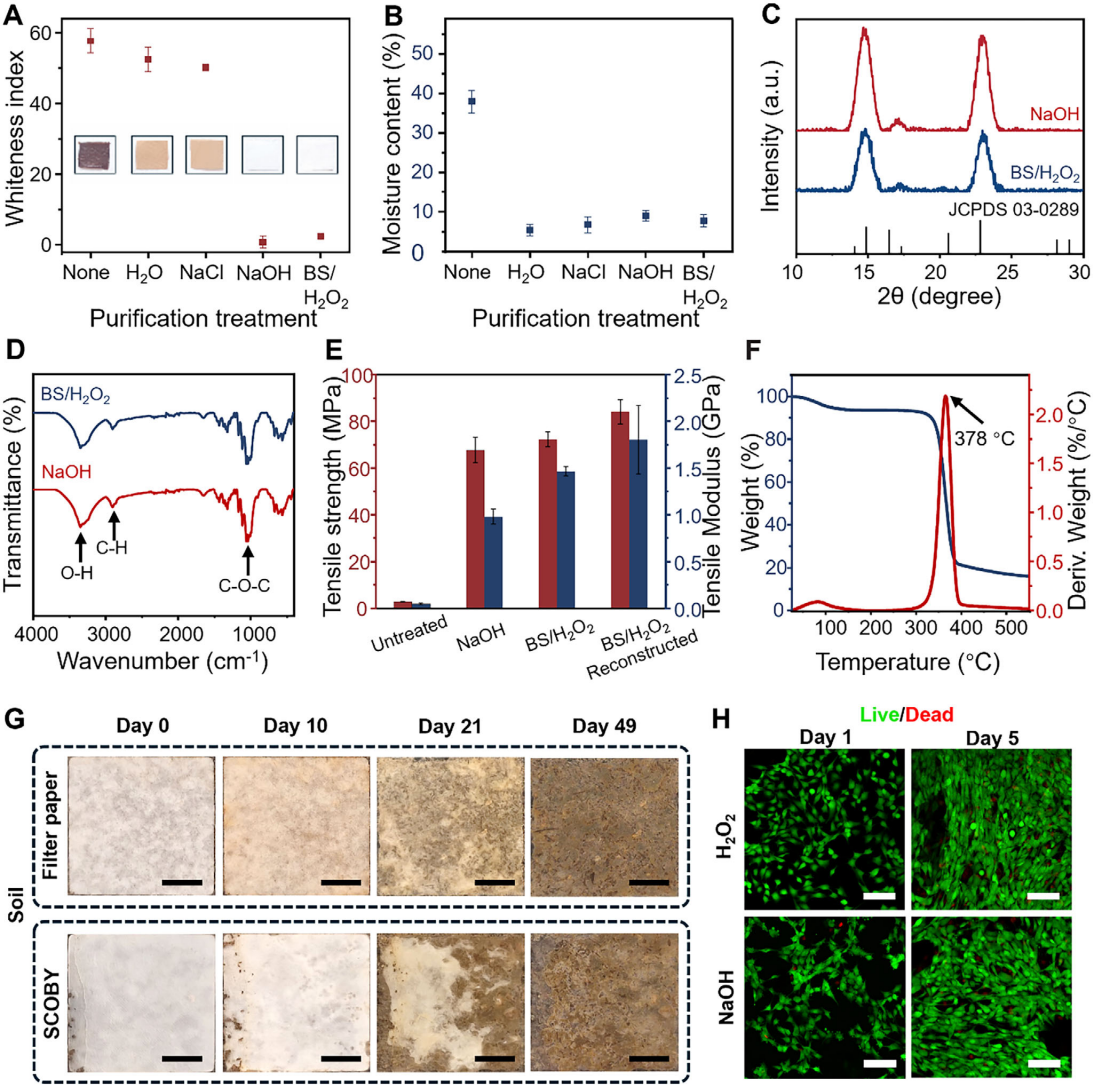
Physical and chemical characterization of KBC. A) Whiteness index of KBC materials purified using various agents. Insets show the corresponding material morphologies (n = 6). B) Moisture absorption of KBC materials over one week, comparing different purification treatments. C) XRD analysis of KBC purified by BS/H_2_O_2_ and NaOH. D) FTIR analysis of KBC purified by BS/H2O2 and NaOH. E) Mechanical performance comparison among untreated, treated, and reconstructed KBC samples (n = 3). F) TGA of a KBC film. G) Soil biodegradation comparison between KBC and filter paper over 49 days. Scale bar: 0.5 cm. H) Live/dead fluorescence staining images of KBC purified with H_2_O_2_ and NaOH. Scale bar: 100 µm.

The performance of various purification agents, including water, sodium chloride (NaCl), NaOH, and a sustainable process using sodium bicarbonate and hydrogen peroxide (BS/H_2_O_2_), was systematically evaluated (**Figure** **2**A). A key parameter in assessing material quality is the whiteness index, which quantifies whiteness relative to standard white paper and is commonly used in the characterization of cellulose‐based materials. Unpurified SCOBY exhibited the highest whiteness index of 57.7 and a visible reddish‐brown hue that darkened over time (Figure 2A inset; Figure S3, Supporting Information), along with the formation of small internal air pockets. This discoloration and instability are attributed to ongoing metabolic activity from residual bacteria and yeast embedded within the material. Simple drying, even at elevated temperatures, failed to sterilize the biologically active pellicle, highlighting the need for effective purification. Purification is essential not only for achieving optical and structural uniformity but also for ensuring the physical and thermal stability of the material. Moreover, thorough removal of residual biological material is critical for preventing potential corrosion of metallic components when KBC is used in electronic devices.

KBC treated with water and NaCl retained a brownish‐yellow color and relatively high whiteness indexes of 52.4 and 50.1, respectively, indicating insufficient removal of impurities. In contrast, only the BS/H_2_O_2_ and NaOH treatments resulted in significantly lower whiteness indexes, 2.30 and 0.69, respectively, suggesting superior purification effectiveness and greater material purity. In addition to whiteness, moisture absorption was evaluated after one week under ambient conditions (Figure 2B). High moisture uptake is typically associated with hygroscopic residues, such as residual sugars, which is undesirable, as it compromises the mechanical stability of the material. Untreated SCOBY showed a moisture absorption rate of 37.9%, while KBC films treated with water, NaCl, BS/H_2_O_2_, and NaOH exhibited reduced moisture uptake of less than 10%. These results underscore the importance of proper purification in enhancing the durability and functionality of KBC for practical applications.

Purification studies confirmed that both BS/H_2_O_2_ and NaOH treatments effectively removed impurities from the SCOBY material, with no residual microbial activity or metabolization observed, indicating successful sterilization. In the BS/H_2_O_2_ method, BS serves as a mild, environmentally friendly base that complements the oxidative action of H_2_O_2_. It establishes a mildly alkaline environment that denatures microbial proteins, disrupts loosely bound organic residues, and loosens the SCOBY matrix, thereby enhancing the penetration and reactivity of H_2_O_2_.

Compared to strong bases like NaOH, BS offers a gentler alternative that preserves the nanofibrous structure of bacterial cellulose while maintaining effective sterilization and impurity removal. This synergistic approach produces a clean, stable KBC film using safe, biodegradable reagents well‐suited for sustainable material processing. Both BS and H_2_O_2_ readily degrade into water, oxygen, and carbon dioxide, underscoring the environmental advantages of this method. While their combination is widely used in medical and household disinfection, its application in cellulose purification remains largely unexplored, highlighting its potential as a green, scalable strategy for producing biocompatible and biodegradable cellulose‐based materials.

X‐ray diffraction (XRD) analysis showed that KBC produced from the treatment of BS/H_2_O_2_ and NaOH both exhibited sharp peaks at 14.4°, 16.8°, and 22.5° (Figure 2C). These diffraction peak angles match the interplanar distance characteristic of the crystalline structure in type Iα and Iβ cellulose. The diffraction peak at 15° corresponds to the (010) plane of cellulose Iα, the (110) plane of cellulose Iβ. Whereas, the peak at 22.5° is associated with the (110) plane of cellulose Iα and the (200) plane of cellulose Iβ.^[^ ^14^ ^]^ Figure 2D shows identical Fourier transform infrared spectroscopy (FTIR) bands for the BS/H_2_O_2_ and NaOH samples. The spectrum profile aligns with previous literature, featuring characteristic bands at 3400 cm^−1^ representing O‐H stretching, 2900 cm^−1^ representing C‐H stretching, and intense bands ≈1100 cm^−1^ representing the presence of C‐O‐C and C‐O bonds, indicating a similar chemical structure as the bacterial cellulose reported in previous studies.^[^ ^15^, ^16^ ^]^ The XRD and FTIR results confirm that the KBC purified using the BS/H_2_O_2_ method retains the expected crystalline and chemical structure of BC, demonstrating its effectiveness as a green alternative to conventional purification methods.

The natural growth of SCOBY pellicles often results in uneven thickness, leading to physical inhomogeneity and mechanical weakness (Figure S4, Supporting Information). To address this, we took inspiration from traditional papermaking technology by developing a reconstruction process comprising pulping, purification, sheet‐forming, and heat‐pressing. This method improves film uniformity and enables precise control over thickness. Mechanical testing (Figure 2E) showed that untreated SCOBY exhibited a low ultimate tensile strength (UTS) of 2.1 MPa and a Young's modulus of 0.05 GPa, indicating weak mechanical performance. Purification significantly enhanced both strength and stiffness. With conventional NaOH treatment, the UTS and Young's modulus increased to 67.7 MPa and 0.98 GPa, respectively. The BS/H_2_O_2_ treatment further improved UTS to 72.2 MPa and increased the Young's modulus to 1.46 GPa.

These improvements are attributed to the removal of unmetabolized sucrose, a hygroscopic compound in untreated SCOBY that readily absorbs moisture from the environment. The absorbed water disrupts hydrogen bonding between cellulose nanofibers, severely weakening the material. Purification eliminates this sucrose, reducing moisture absorption and allowing the KBC to maintain its mechanical integrity. We further demonstrated that the reconstruction process can be repeated to enhance mechanical properties. After four cycles, the UTS and Young's modulus slightly increased to 84.1 MPa and 1.8 GPa, respectively. Therefore, the BS/H_2_O_2_ method presents a greener alternative to NaOH bleaching without compromising mechanical performance, and it opens new opportunities for recycling KBC films through repeated pulping and film‐forming processes.

To verify the integrity of the material under high humidity over time, mechanical tests on freshly prepared material as well as samples stored under laboratory conditions (80% RH, 25 °C) for one and two months were conducted, as shown in Figure S5 (Supporting Information). The tensile strength showed only a slight decrease from 87.2 ± 8.7 MPa (fresh) to 79.3 ± 22.4 MPa (1 month) and 78.3 ± 23.7 MPa (2 months). The elastic modulus remained stable, with values of 1.30 ± 0.11 GPa, 1.23 ± 0.10 GPa, and 1.28 ± 0.07 GPa, respectively. These results indicate that storage under ambient laboratory conditions does not significantly alter the mechanical strength of the material, despite its potential for moisture uptake from the environment. This observation was further supported by one‐way ANOVA tests, which yielded p = 0.83 for tensile strength and p = 0.71 for elastic modulus, confirming no statistically significant differences as *p* >0.05 between freshly prepared samples, samples stored for one month, and samples stored for two months.

Figure 2F presents the thermogravimetric analysis (TGA) of the KBC film, revealing distinct thermal behavior characteristic of cellulose‐based materials. A sharp peak in the derivative weight curve at 378 °C corresponds to cellulose decomposition, which occurs within the 300–400 °C range. An initial weight loss near 100 °C is attributed to the evaporation of residual moisture. The final residue, comprising incombustible ash, accounts for ≈20% of the original mass. The high thermal degradation temperature underscores the suitability of KBC for electronic applications, which typically operate at temperatures below 125 °C.^[^ ^17^ ^]^

Biodegradation tests further confirmed the environmental compatibility of KBC. As shown in Figure 2G, KBC exhibited high degradability in soil, comparable to that of filter paper. Over 49 days, both materials underwent significant morphological changes and browning, indicating progressive decomposition. While the degradation rate of KBC was slightly slower than that of filter paper, this is attributed to its higher crystallinity, which can hinder microbial access to the cellulose backbone. Nonetheless, soil‐borne microorganisms were able to effectively biodegrade KBC, demonstrating its environmental degradability despite its crystalline structure.

To study biocompatibility, KBC films were seeded with C2C12 mouse myoblast cells. The cells adhered well to the film surface, indicating that KBC supports cell attachment and viability. After 5 days of culture, the KBC films treated with either H_2_O_2_ or NaOH exhibited high cell viability of 94.9% and 94.4%, respectively. Live/dead fluorescent staining images (Figure 2H) corroborate these results, confirming that the material is non‐cytotoxic and suitable for applications involving direct contact with living cells or organisms. This also suggests that the material is biocompatible when released into the natural environment, posing minimal risk to surrounding biological systems.

To highlight the competitiveness of KBC as a substrate for biodegradable sensors and electronics, we systematically compared its mechanical properties and degradation behavior with other commonly used biodegradable materials, including paper,^[^ ^18^ ^]^ polylactic acid (PLA),^[^ ^19^, ^20^, ^21^ ^]^ silk composites,^[^ ^22^ ^]^ and starch^[^ ^23^ ^]^ (Table S1, Supporting Information). KBC exhibits a tensile strength of 70–100 MPa, which is comparable to paper (60–100 MPa) and higher than PLA (40–85 MPa), silk composites (15–40 MPa), and starch (6–12 MPa). Its Young's modulus (1–2 GPa) provides a balance between flexibility and stiffness, lying between the high rigidity of paper (6–8.5 GPa) and the low stiffness of starch (0.1–0.9 GPa) or silk composites (0.4–1.5 GPa). Importantly, KBC degrades within ≈49 days, offering a practical timescale that is faster than PLA (6 months–2 years) while maintaining greater stability than silk composites (5 min in hot water) or starch (30 days in soil). This unique combination of mechanical robustness, moderate flexibility, and controlled biodegradation positions KBC as a promising and competitive substrate for the development of sustainable, transient electronic devices.

Additionally, the KBC film surface exhibits a microscale texture with nearly identical root‐mean‐square roughness (Rq) values in the horizontal (5.57 µm) and vertical (5.69 µm) directions, indicating a uniform surface across the KBC films (Figure S6, Supporting Information). Conductive traces and electronic components can be integrated onto the KBC films for the fabrication of various sensing and electronic devices. Gold was selected as the conductive material due to its excellent electrical conductivity, chemical stability, biocompatibility, and widespread use in electronic applications.^[^ ^24^ ^]^ Compared with biodegradable metals, such as magnesium and zinc, that corrode rapidly under high humidity, sputtered gold remains stable and, during cellulose degradation, fragments into particles that disperse harmlessly into the environment.^[^ ^25^, ^26^ ^]^ Carbon‐based materials (e.g., graphene, carbon nanotubes)^[^ ^27^, ^28^ ^]^ and conductive polymers (e.g., PEDOT: PSS)^[^ ^29^ ^]^ represent promising alternatives for conductive traces; however, their technological maturity and reproducibility remain limited compared to gold, constraining their applicability in reliable device fabrication. In addition, gold is a naturally occurring element found in soil at varying concentrations,^[^ ^30^ ^]^ and although it is not biodegradable, it can be recovered or recycled from the substrate, potentially minimizing environmental impact when combined with the biodegradable KBC platform.

**Figure 3:**
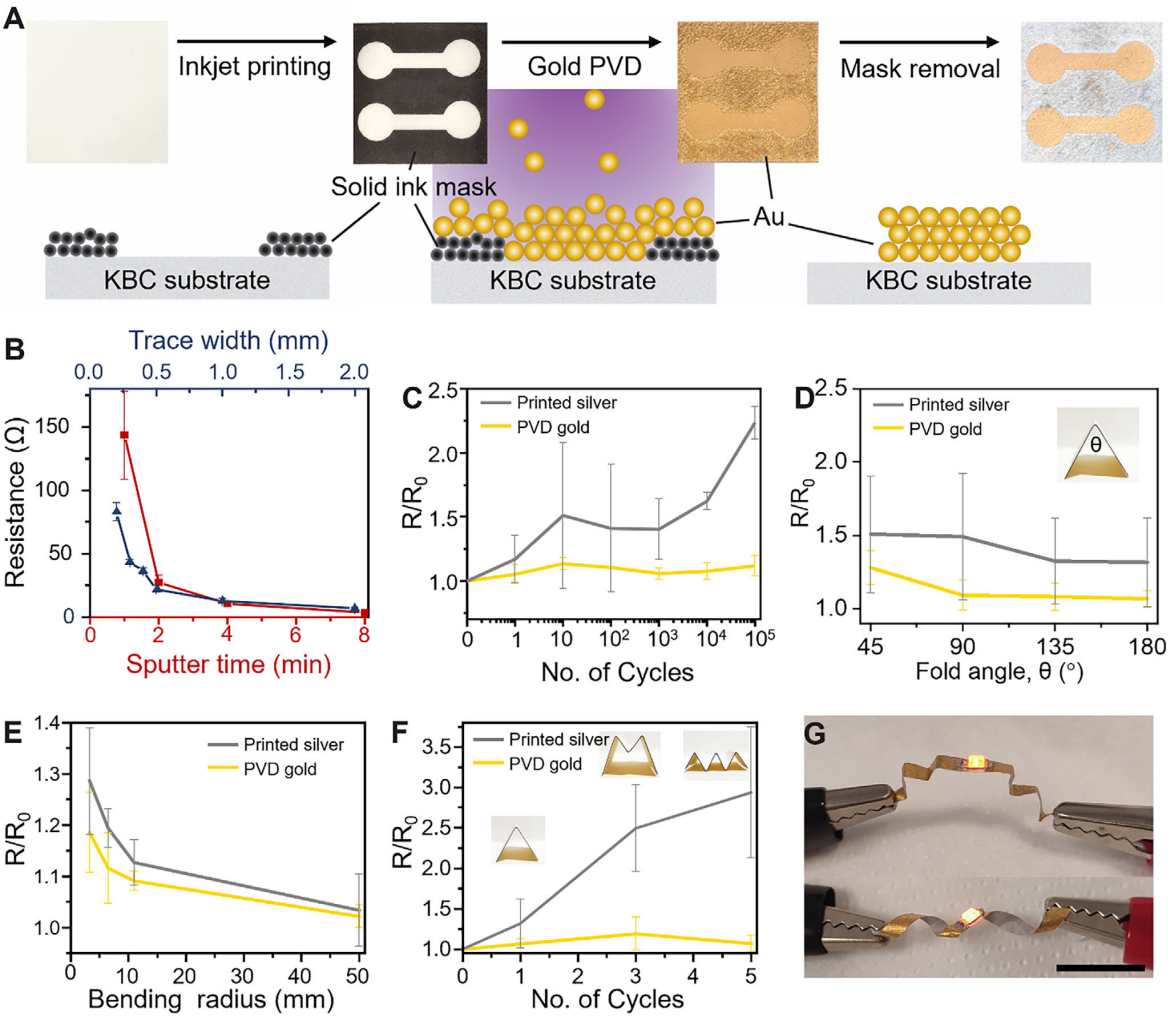
Characterization of gold circuit patterning on KBC substrates. A) Schematic illustration of the fabrication process for gold circuits on KBC films, involving inkjet printing of a negative mask, gold sputtering, and mask removal via ultrasonication. B) Resistance of gold circuits as a function of trace width and sputtering time (n = 3). C) Relative resistance change of conventional printed silver and gold PVD circuits under repeated bending cycles (n = 3). D) Resistance recovery of initially folded circuits as they are gradually straightened (n = 3). E) Relative resistance change of circuits under varying bending radii (n = 3). F) Relative resistance change of circuits after repeated folding cycles (n = 3). G) Optical images of an SMD LED mounted on a gold PVD‐patterned KBC device, demonstrating excellent conductivity retention after multiple bends and folds. Scale bar: 1 cm.

KBC serves as a promising substrate for electronics owing to its low and stable dielectric constant of ≈3 at frequencies above 10 Hz (Figure S7, Supporting Information), which helps minimize signal loss and crosstalk. To fabricate conductive patterns, we developed a process that begins with inkjet printing a negative pattern using an ink mask directly onto the KBC substrate. This is followed by gold sputtering, a form of physical vapor deposition (PVD), and subsequent mask removal using ultrasonication, leaving behind well‐defined gold circuits (**Figure** **3**A).

As shown in Figure 3B, increasing sputtering time significantly reduced the resistance of the conductive paths. Measured resistances were 143, 27, 10.7, and 3.3 Ω for sputtering durations of 1, 2, 4, and 8 min, respectively, attributed to improved connectivity between gold particles with longer deposition times. Notably, rotating the sample by 90° or 180° had no measurable effect on the deposition quality, demonstrating the spatial robustness of the PVD process. Reducing the line width of the printed traces led to a steep increase in resistance. Specifically, line widths of 0.2, 0.3, 0.4, 0.5, 1, 2, and 5 mm exhibited resistances of 82.7, 43.5, 36.3, 22.0, 12.7, 6.8, and 3.1 Ω, respectively. The nonlinear resistance trend is likely due to the increased influence of microstructural defects and discontinuities in narrower sputtered lines.

These imperfections play a more dominant role in thinner traces, limiting electrical performance. Using this method, we achieved reliable line widths ranging from 0.3 to 1 mm on the KBC substrate. This range of widths was chosen to match the widths of the conductive traces in conventional printed circuit boards. The printed traces were slightly wider than expected, as summarized in Table S2 (Supporting Information), likely due to limitations in the precision of the inkjet printer being used. From Figure S8 (Supporting Information), each printed line exhibited clearly defined borders, confirming that surface roughness does not compromise the fidelity of circuit patterning.

We compared the mechanical and electrical durability of gold circuits fabricated via sputtering with silver circuits fabricated using conventional printing techniques. As shown in Figure 3C, resistance measurements over 10^5^ folding cycles revealed a significant difference in performance between the two. The resistance of the printed silver circuit increased by 2.23 times, whereas the sputtered gold circuit exhibited only a marginal increase of 1.11 times, indicating superior stability of the latter under repeated mechanical deformation. In addition, SEM imaging (Figure S9, Supporting Information) shows that gold can be uniformly coated onto the KBC substrate despite the inherent surface roughness of the material. After repeated bending tests, fold lines were observed, but no flaking or delamination occurred, indicating good adhesion of the gold layer to the substrate.

To further investigate resistance recovery, we monitored the change in resistance during a single folding‐unfolding cycle. The circuits were folded and then gradually unfolded in 45° increments until fully straightened (Figure 3D). The resistance returned to 1.31 and 1.06 times the initial value for the printed silver and sputtered gold circuits, respectively. We also investigated the effect of bending radius on circuit resistance. At radii of 3.3, 6.5, 11, and 50 mm, the printed silver circuit exhibited resistance increases of 1.29, 1.19, 1.13, and 1.03 times its original value, while the sputtered gold circuit showed increases of 1.19, 1.11, 1.09, and 1.02 times, respectively (Figure 3E). These results demonstrate the superior mechanical resilience of gold‐sputtered circuits in flexible and foldable electronic applications.

Furthermore, we studied the cumulative effect of folding. After five folds, the resistance of the printed silver circuit increased dramatically to 2.94 times its initial value, with huge variations among samples. At the same time, the sputtered gold traces remained below 1.16 times throughout (Figure 3F). This highlights the robustness of gold sputtering for flexible device applications.

Gold sputtering creates a compact, uniform, and well‐adhered conductive layer with a low surface‐to‐volume ratio. During sputtering, high‐energy gold atoms are deposited onto the KBC substrate, promoting strong adhesion and dense film formation. In contrast, printed silver inks typically contain polymeric binders that are mechanically weaker and more prone to delamination or cracking under repeated stress. As a result, the printed silver traces are more susceptible to microfracture‐induced material fatigue, leading to a sharp increase in resistance over time.

To demonstrate practical functionality, we integrated a surface‐mount device (SMD) light‐emitting diode (LED) onto a gold‐sputtered KBC film. Even after multiple folds and bends, the LED remained functional, confirming the mechanical and electrical reliability of gold‐sputtered traces on KBC substrates for use in flexible electronic devices (Figure 3G).

To assess the long‐term electrical stability of gold‐sputtered KBC under high humidity, resistance measurements of gold traces on the KBC substrate were performed over a two‐month storage period at 80% RH (Figure S10, Supporting Information). The resistance values show only minor fluctuations throughout the monitoring period, with no systematic increase or decrease that could be attributed to moisture uptake. This stability demonstrates that the KBC paper produced using the method described in this study is not significantly affected by high ambient humidity. In particular, the absence of resistance drift suggests that the substrate does not undergo swelling or dimensional changes that could compromise the electrical performance of printed gold tracks. These findings confirm the suitability of the KBC substrate for applications requiring reliable electrical conductivity under humid environmental conditions.

To evaluate the biodegradability of gold‐sputtered KBC, a mock electronic device was buried in soil alongside a *Vigna unguiculata unguiculata* plant and monitored over ≈2 months (Figure S11, Supporting Information). Within just 10 days, the KBC substrate began to degrade, as evidenced by a noticeable color change from white to translucent, indicating rapid microbial decomposition. Although the gold traces remained visible due to their non‐biodegradable nature, gold is bioinert and did not hinder plant growth. After nearly two months, the plant showed healthy development with no signs of stress or growth inhibition, suggesting that the degradation products of the KBC substrate are environmentally benign and do not negatively impact surrounding biological systems.

**Figure 4:**
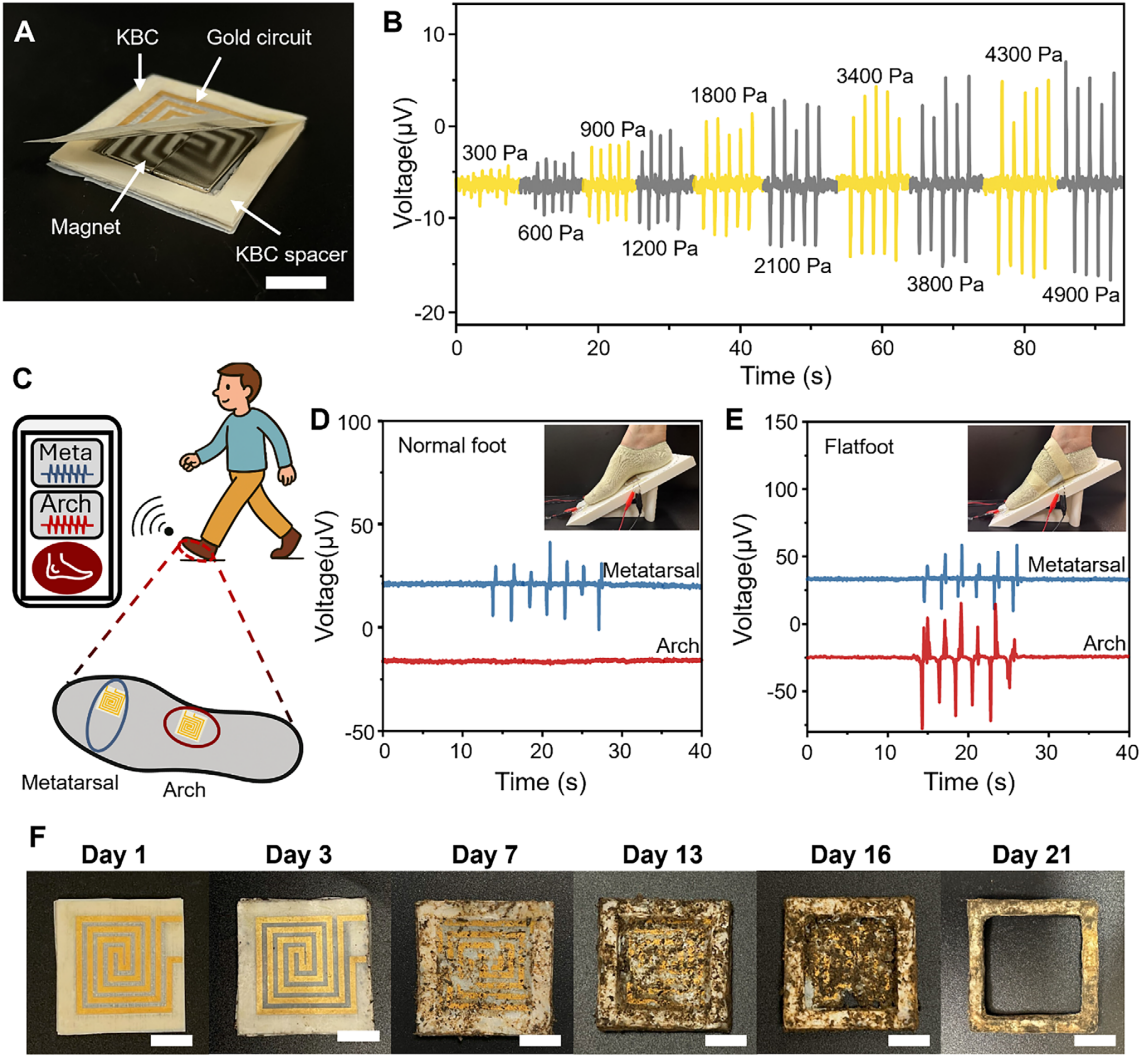
Design, performance, and biodegradation of the KBC‐based pressure sensor. A) Optical image of the KBC‐based pressure sensor, consisting of three layers: a top KBC sheet with a gold‐sputtered coil, a 1.5 mm‐thick square hollow KBC spacer anchoring a 1.0 mm‐thick magnet, and a bottom KBC film supporting the magnet. Scale bar: 1 cm. B) Sensor response under varying applied pressures. C) Schematic illustration of the self‐powered system for flat foot monitoring. Sensor response curves during stepping for D) a normal foot and E) a flat foot. Inset scale bars: 5 cm. F) Time‐lapse images showing the biodegradation of the pressure sensor in soil. Scale bar: 1 cm.

The growing demand for sustainable health‐monitoring electronics has driven significant interest in biodegradable materials and wearable sensing technologies. Foot pressure monitoring plays a crucial role in assessing gait and detecting abnormalities such as flatfoot.^[^ ^31^ ^]^ We applied the gold‐sputtered KBC circuit in a self‐powered pressure sensor for real‐time flatfoot condition monitoring (**Figure** **4**A). The device operates on the principle of electromagnetic induction. Applying pressure on the sensor's surface alters the interactive distance between the gold coil and an integrated magnet, consequently changing the magnetic flux across the coil and generating a measurable voltage output. We evaluated its sensing performance under different pressures at a consistent loading speed of 1 mm s^−1^. As shown in Figure 4B, the voltage output exhibited a direct correlation with increasing loading pressure, demonstrating a broad sensing range from 300 to 4900 Pa. As illustrated in Figures S12 and S13 (Supporting Information), the KBC‐based pressure sensor exhibits a high linearity (R^2^ = 0.993) and a fast response time of 25.4 ms, comparable to recently reported self‐powered flexible pressure sensors.^[^ ^32^, ^33^, ^34^, ^35^, ^36^ ^]^ Remarkably, it consistently displayed repeatable and stable responses at each pressure level, highlighting its reliability for continuous monitoring applications. This robust performance is attributed to the mechanical and electrical stability of the KBC film and the low resistance of the gold‐sputtered conductive traces, which remain functional even under bending and deformation.

We further explored the potential of the pressure sensor for flatfoot monitoring (Figure 4C). In our setup, a 3D‐printed slope was employed, and two sensors were fixed on the slope underneath the arch and the 2nd and 3rd metatarsal areas of the foot. The heel remained in contact with the slope, allowing the foot‐slope contact area to progressively extend from heel to toe, thereby mimicking the natural walking gait. To simulate a flat foot, a silicone pad was fixed at the arch of a normal foot. For the normal foot, the sensor located under the metatarsal region displayed reproducible responses to foot stepping. No apparent voltage signals were observed from the arch sensor as it did not come into contact with the foot arch (Figure 4D). For the flatfoot case, the metatarsal sensor recorded lower voltage outputs, with the peak signal measuring ≈22% less than that of the normal foot. This reduction is due to a more uniform pressure distribution across the entire foot. The arch sensor recorded an obvious voltage as there is contact with the foot arch (Figure 4E). By comparing the outputs from both sensors, the system effectively distinguishes between normal and flatfoot conditions, enabling real‐time diagnostic monitoring.

The overall design of the pressure sensor aligns with the principle of sustainability, minimizing environmental impact throughout its lifecycle. To evaluate its environmental performance, we disassembled the sensor, reused the magnet, and studied the biodegradation of the remaining components in soil under simulated rainfall conditions (15 mL of water added daily). As shown in Figure S14 (Supporting Information), the sensor's mass initially increased during the first 16 days due to water absorption. This was followed by a sharp decrease in mass as microbial degradation of the KBC substrate progressed. By day 21, both the circuit and bottom layers had fully disintegrated (Figure 4F). The sensor's high sensing performance, mechanical durability, and biodegradability underscore the significant potential of KBC as a sustainable substrate for disposable health‐monitoring wearable devices. This material, derived from tea‐fermenting waste byproduct, paves the way for next‐generation eco‐friendly electronics, seamlessly integrating performance with environmental responsibility.

## 3 Conclusion

We developed an eco‐friendly SCOBY pulping, purification, and sheet‐forming process to fabricate white KBC for sustainable electronics. This process begins with SCOBY, the waste byproduct of kombucha tea fermentation, which contains living microorganisms and is unsuitable for electronic applications in its raw form. Our method produces a uniform, stable, and biodegradable KBC film by utilizing BS/H_2_O_2_, effectively sterilizing, whitening, and removing unstable compounds while avoiding the conventional use of strong base NaOH. The KBC films could also be recycled by reconstructing them, further advancing the sustainability and circularity of the material. TGA, XRD, and FTIR analyses confirmed that the resulting material exhibited high purity and a significant degree of crystallinity, with no unexpected changes to its chemical composition post‐purification and processing. Tensile tests indicated that the reconstructed material demonstrated superior mechanical properties compared to the as‐grown sheets. For fabricating conductive patterns, we developed a process that begins with inkjet printing a negative pattern directly onto the KBC substrate, followed by gold sputtering and subsequent mask removal, resulting in well‐defined gold circuits. These circuits exhibited low resistance and robust electrical properties, even under sharp folding angles, numerous folds, and repeated folding cycles. We applied this technology to a pressure sensor for flatfoot measurement. The KBC‐electronic demonstrated excellent biodegradability in soil without negatively impacting plant growth. The demonstrated versatility, efficiency, and eco‐friendly attributes highlight the importance of continued exploration and development of KBC as a valuable and sustainable material for electronics.

## 4 Experimental Section

### Materials

All chemicals were used as received without further purification. Kombucha starter tea was procured from a local commercial kombucha drink company (Original Komby, Kombynation, Singapore), and subsequent batches of tea were brewed using black tea (Lipton, Singapore) as the hydrogen source and sucrose (Macklin, China) as the carbon source. NaCl (Sigma–Aldrich) dissolved to 0.9% concentration, NaOH (Sigma–Aldrich) dissolved to 1 m concentration, sodium bicarbonate (baking soda, BS) (Sigma–Aldrich) dissolved to 1 m concentration, and hydrogen peroxide (3% H_2_O_2_, Sigma–Aldrich) diluted to 0.5% in deionized water were used in purification.

### Kombucha Brewing and SCOBY Growth

A set of three tea bags, each containing 2 grams of tea, was added to 1.5 L of boiling deionized water and left to steep for 20 min. After which, the tea bags were removed, and 250 g of sugar was added. The sweetened tea was stirred for the sugar to fully dissolve and cool to below 30 °C before adding 750 g of starter Kombucha tea. The kombucha was then incubated in a glass tray (35 × 25 cm^2^ MIXTUR Oven/serving dish, IKEA, Singapore) to maximize the area of the liquid‐air interface and improve SCOBY yield. The trays were covered with an air‐permeable non‐woven sheet to prevent contamination and left to incubate at 25 °C. SCOBY was harvested twice, at the end of the first and second week of incubation, after which the kombucha was left to age to pH 2.4 for at least 2 more weeks, before being reused as starter tea. The harvested SCOBY was quickly rinsed with deionized water to remove yeast strands and patted dry with tissue before use.

### KBC Purification Methods

Conventional cleaning treatments using 1 m NaOH solution, 1% NaCl solution, and H_2_O were used to treat the harvested KBC. NaOH and NaCl solutions were prepared by dissolving the salts as purchased in the required quantity into purified water. The samples were immersed in a surplus of the respective solutions for 1 h, followed by a soak in water for 1 week, with water changes on days 3, 5, and 6. As for treatments with BS/H_2_O_2_, 400 g of SCOBY pellicle was blended with 400 g of deionized water for 2 min on low speed (Morris MS‐BL250). 38.3 g of BS and 112.5 g of H_2_O_2_ were added, and the mixture was stirred for another 25 min before draining thoroughly to ≈40 g. The KBC was then neutralized by stirring and rinsing 3 times in 1800 g of deionized water. Each round of stirring was 20 min long. After neutralization, the BC is dried at 50 °C and stored.

### Whiteness Index Calculation

The degree of whiteness of the KBC films was measured by taking a photo of all the samples against a sheet of white printing paper in the background. The CIE Lab color coordinates on each sample were obtained using a color picker, and the whiteness was calculated by Equation (1):

(1) $$\mathrm{Whiteness}\;\mathrm{index}\;=WI_{\mathrm{white}\;\mathrm{paper}}-0.511L^{*}-2.324a^{*}-1.100b^{*}$$

where WI_white paper_ is the whiteness index of a regular sheet of white printing paper, and L*, a* and b* are the color coordinates of the samples. Each sample was measured at six different points to give an average value.

### Reconstructing KBC Films from Blended Pulp

Dried KBC, weighing 0.8 grams, was blended with 150 g of deionized water on low for 2 min. Then, 200 g more deionized water was added before pouring the blend into an 11 cm square mold placed on a fine mesh and foam to allow drainage of excess deionized water. After sitting for 15 min, the frame was removed and the KBC was left in an oven overnight at 50 °C to dry completely. The dried sheets were straightened by dipping them in deionized water for 5 min and heat‐pressing (Cooldiy Digital Graphic Co., Ltd., China) at 125 °C for 120s. Subsequent samples were cut from the straightened films into different geometries as required.

### General Characterization

Uniaxial tensile characterization was performed using the Zwick Roell Z2.5 Materials Testing Machine. The samples were cut into dogbone shape according to ASTM D638 Type V specifications for testing. FESEM images were taken on a Hitachi S‐4300. XRD tests were conducted using Shimadzu XRD‐6000. FTIR tests performed using Bruker Vertex 80v. TGA tests were performed using TA Instruments Q500 at a ramp rate of 20 °C min^−1^. Dielectric constant was characterized using Novocontrol Technologies Alpha‐A high‐performance frequency analyzer.

### Biocompatibility Test

The biocompatibility of KBC films was evaluated by culturing C2C12 murine myoblast cells on 2 mm square samples. The films were sterilized by immersion in 70% ethanol for 30 min, air‐dried under sterile conditions, and placed in a 24‐well plate. Cells were seeded using 50 µL of suspension on each film, at a density of 1000 cells mm^−^^2^, and incubated for 4 h to allow attachment. Afterward, 1 mL of complete growth medium (DMEM supplemented with 10% FBS and 1% penicillin‐streptomycin) was added, and the medium was changed daily. Cell viability was assessed on days 1 and 5 using LIVE/DEAD staining (calcein‐AM and ethidium homodimer‐1; ThermoFisher), and samples were imaged using a Zeiss LSM 700 laser scanning confocal fluorescence microscope.

### Gold Circuit Fabrication

Conductive pathways were introduced to the cellulose substrate by initially printing the negative pattern as a mask using an office paper printer (HP OfficeJet Pro 9015e). The masked substrates were then sputter‐coated with gold for 480s (Cressington 108 Auto Sputter). To remove the mask, the sputtered samples were immersed in a surplus of acetone for 15 min, followed by sonication at 37 Hz for 20s. Afterward, it was lightly patted dry with tissue and oven‐dried for 1 h.

### Characterization of the Gold‐Sputtered Circuit

The resistance of conductive pathways was measured using the Keithley DMM7510 7½ Digit Graphical Sampling Multimeter. Circuit lines with a width of 0.5 cm and a length of 2 cm were tested for resistance, with measurement probes placed at the ends of the lines. Resistance measurements were taken in triplicate on parallel lines printed. The test was repeated with varying line widths of 0.1, 0.2, 0.3, 0.4, 0.5, 1, 2, and 5 mm, as well as with folding the material at fold angles (θ) of 90° and 180° midway of the path on 5 mm wide lines. The resistance was measured while holding the material at the respective θ. The smallest possible distance between each line was found by testing for short circuit between 0.3 mm lines spaced different distances apart, at 0.1, 0.2, 0.3, and 0.4 mm. The folds were created by folding the samples 180°, then relaxing the fold and measuring the resistance with the fold angle held at θ ≈120°. Cyclic tests were performed on a linear motorized actuator moving back and forth to fold and unfold the sample with a θ of 180° at 5 Hz.

### Surface Roughness Measurement

Surface roughness of KBC films was measured using a contact‐mode surface profilometer (Nano Scratch Tester, CSM Instruments, Switzerland) equipped with an Al_0.5_CoCrFeNi alloy tip (tip radius: 2 µm). During the measurement, a constant normal force of 0.1 mN was applied to maintain stable contact between the tip and the sample surface. The probe was scanned across the sample with a lateral speed of 5 mm min^−1^ over a total scan length of 1 cm. To improve reliability, three parallel lines were scanned in the horizontal and vertical directions, respectively, with each line separated by 0.5 cm. The obtained height profiles were subsequently analyzed to extract the root‐mean‐square roughness (Rq) values.

### Biodegradation

Biodegradation in soil was conducted using 1.8 cm square samples with a thickness of ≈0.2 mm. Triplicates were buried under 5 cm of potting soil (GreenSpade, Singapore), which was watered with 15 mL of deionized water every week to maintain a 20–50% moisture level throughout the experiment. Degradation samples with gold traces were potted with black eye bean sprouts (*Vigna unguiculata unguiculata*), which had been pre‐sprouted in damp tissue for 3 days.

### Design and Characterization of KBC‐Based Pressure Sensor

The KBC‐based pressure sensor is composed of three layers. The top layer is a piece of KBC coated with a planar gold coil. The middle layer consists of a 1.5 mm‐thick square hollow spacer formed by stacking multiple layers of KBC films, which provides structural support while housing a 1.0 mm‐thick magnet. The magnet enables voltage signal generation upon external pressure in conjunction with the coil above. The bottom is another KBC film that holds the magnet. All layers are integrated using biodegradable plant‐based glue, resulting in a planar pressure sensor with dimensions of 32 mm × 32 mm × 1.6 mm.

The KBC‐based pressure sensor was mounted on a fixed stage of the compression machine (JSV‐H1000, ALGOL INSTRUMENT CO., LTD). A 3D‐printed PLA pillar, with a cross‐sectional area of 18×18 mm^2^, was employed as the indenter to transmit force. The pressure loading was applied to the sensor through the PLA indenter. The indenter was placed in close contact with the top gold coil layer while ensuring zero initial pressure. Different pressure loadings were subsequently applied by controlling the displacement of the indenter. The induced voltage in the coil when pressure is applied was collected via a digital source‐meter (Keithley DMM 7510). By controlling the displacement of the indenter, different pressures were sequentially loaded, enabling quantitative evaluation of pressure‐sensing performance. Induced voltages were generated during both loading and unloading with opposite polarities. The analysis focused on the output signals obtained during the loading process, where the induced voltage exhibits positive polarity.

### Statistical Analysis

All data are presented as mean ± standard deviation (SD), with a sample size (n) of 3 for each measurement. Statistical significance (*p*< 0.05) was determined using one‐way ANOVA, unless otherwise specified in the text.

## Conflict of Interest

The authors declare no conflict of interest.

## Supporting information



Supporting Information

## Data Availability

The data that support the findings of this study are available from the corresponding author upon reasonable request.
