# Supplementary material for: From Grave to Cradle: Kombucha Waste for Sustainable Electronics
Source: Adv Sci (Weinh). 2025 Oct 17;13(15):e14521. doi: 10.1002/advs.202514521 (PMC13042482; doi:10.1002/advs.202514521)
Supplement: Supplementary file 1 — Supporting Information [file ADVS-13-e14521-s001.docx]

Supplementary information for

**From grave to cradle: Kombucha waste for sustainable electronics**

Xin Ying Chan^1^, Xiaolu Sun^1^, Eddy Yi Ler Pang^1^, Iris Zhiyu Ren^1^, Xuan Zhang^1^, Pengyu Chen^1^, Yu Jun Tan^1,*^

^1^Department of Mechanical Engineering, College of Design and Engineering, National University of Singapore, 117575 Singapore

*E-mail: yujun.tan@nus.edu.sg

**The file includes:**

Supplementary Figures S1 to S14

Supplementary Tables S1 and S2

**Figure S1.** Schematic illustrating SCOBY formation through the interaction of yeast, bacteria, and tea compounds in kombucha. During kombucha fermentation, a symbiotic culture of bacteria and yeast (SCOBY) develops at the air–liquid interface of sweetened tea. Yeast cells initiate the process by hydrolyzing sucrose into glucose and fructose, which are subsequently fermented into ethanol and carbon dioxide. These metabolic products alter the local environment and promote the growth of acetic acid bacteria. The bacteria oxidize ethanol into acetic acid and secrete extracellular cellulose nanofibers. These nanofibers assemble into a gelatinous pellicle at the interface, which thickens over time. The coordinated metabolic activities of yeast and bacteria shape both the biochemical conditions and the spatial organization of the cellulose matrix, ultimately leading to SCOBY formation.


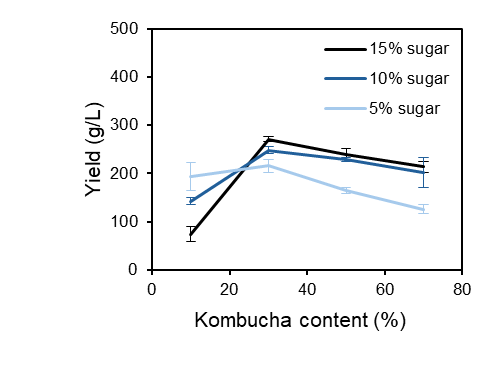


**Figure S2.** Yield of SCOBY pellicle with varying kombucha tea content and sugar concentration (n = 3).

**Figure S3.** Oven-dried, unpurified SCOBY. (a) Picture shows the brown, sticky appearance of the SCOBY, indicating residual biological activity from surviving microorganisms and ongoing fermentation processes, making this material fundamentally unsuitable for electronic substrates. Three critical limitations arise: its inherent instability causes property degradation over time, the persistent microbial activity poses contamination risks, and its moisture sensitivity violates the dry-environment requirements of electronic fabrication. Although cellulose itself acts as an insulator, the unprocessed SCOBY's retained moisture and ionic impurities create electrically unpredictable behavior that may manifest as leakage currents or signal interference in circuits. (b) Picture shows mold proliferation on hydrated SCOBY. This phenomenon demonstrates the material's hygroscopic nature, where ambient water absorption facilitates microbial growth that further compromises structural and electrical reliability in electronic applications.

**Figure S4.** Purified but unreconstructed SCOBY sheet. The black arrow indicates trapped air bubbles that create surface irregularities, resulting in an uneven, non-flat morphology unsuitable for circuit patterning. Unlike reconstructed films from pulping and sheet-forming, this sheet lacks thickness control and exhibits wrinkles (orange arrows), further compromising its utility as an electronic substrate. The inherent surface irregularities and porous nature of unprocessed SCOBY prevent precise deposition of conductive materials or fabrication of microelectronic features, rendering it incompatible with electronic manufacturing processes.


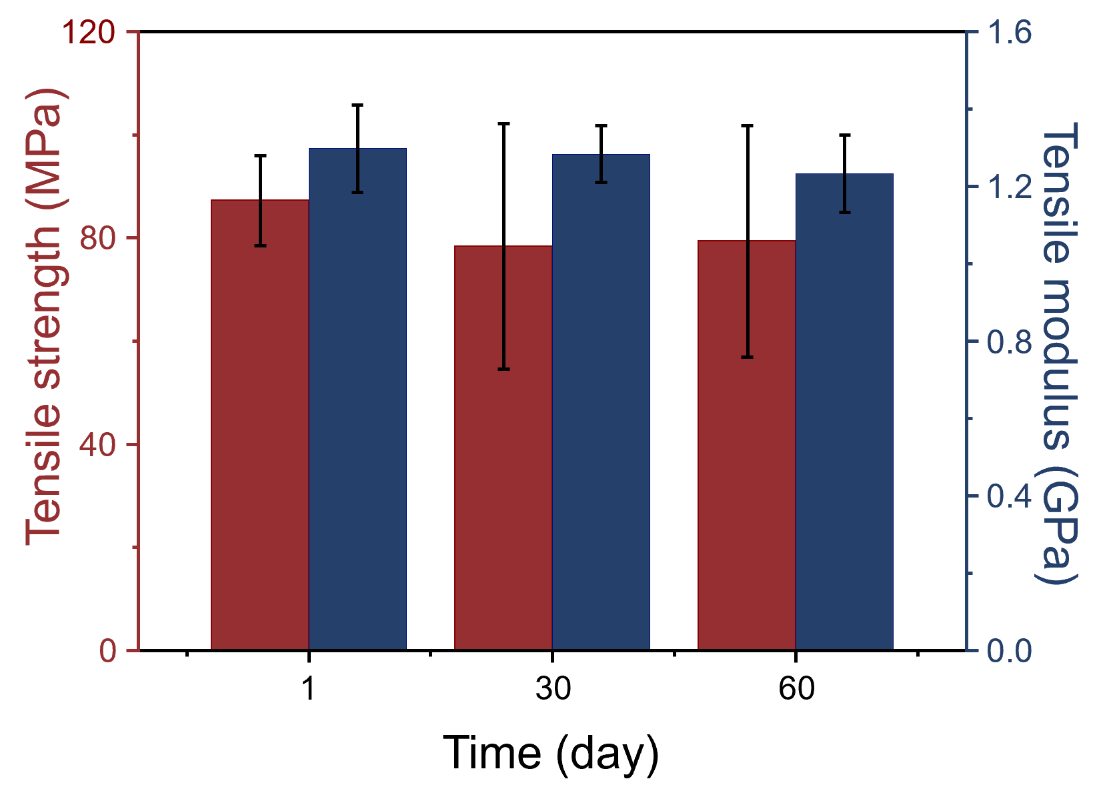


**Figure S5.** Mechanical strength of KBC substrates over two months at room humidity (80% RH).


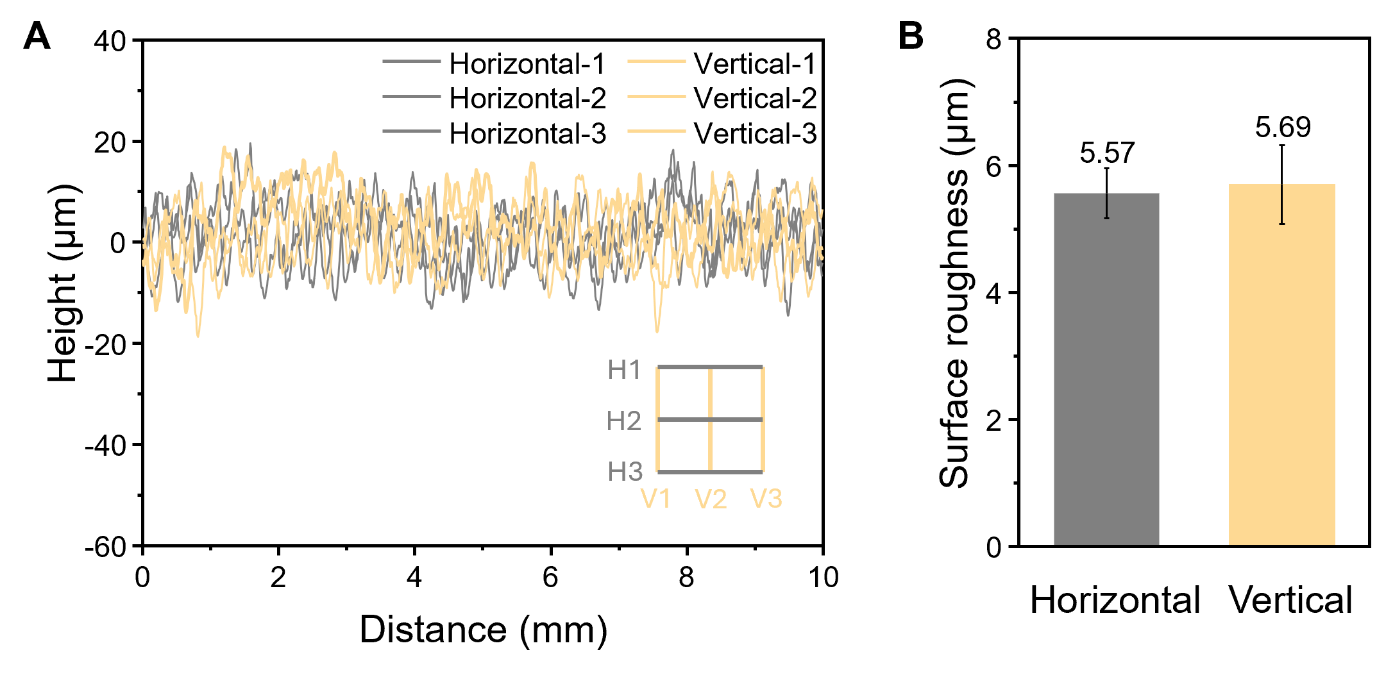


**Figure S6.** (A) Surface height profiles and (B) surface roughness of KBC films.

**Figure S7.** Dielectric constant of KBC film in a frequency sweep.


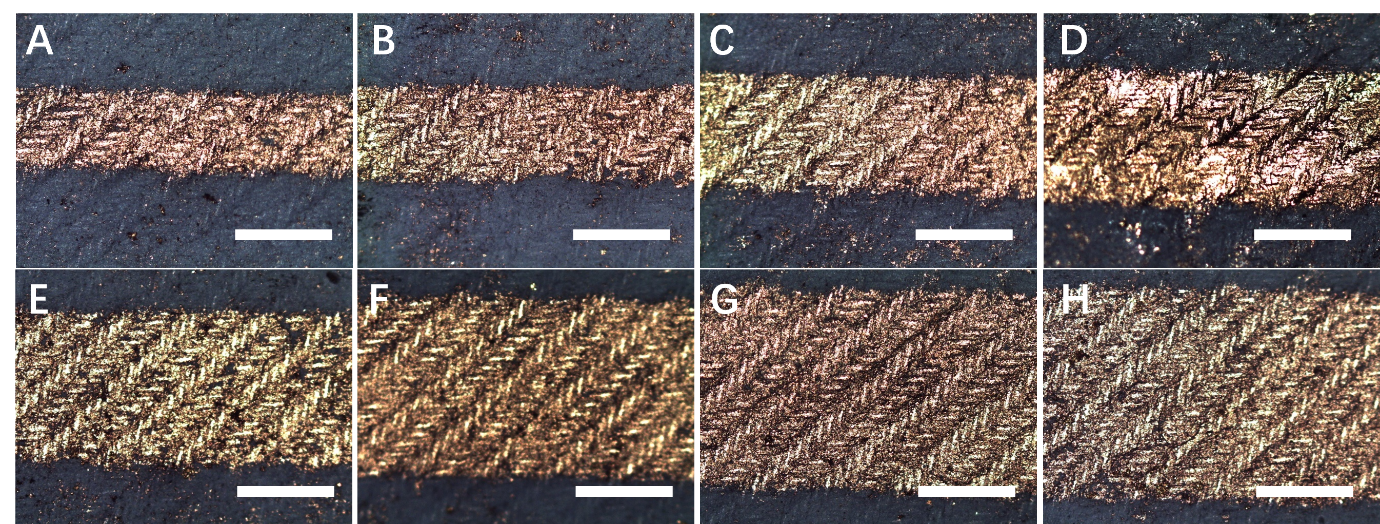


**Figure S8.** Optical photographs of gold circuits with different designed widths ranging from 0.3 mm to 1.0 mm on KBC substrates. Scale bar: 500 µm.

(A) 0.3 mm, (B) 0.4 mm, (C) 0.5 mm, (D) 0.6 mm, (E) 0.7 mm, (F) 0.8 mm, (G) 0.9 mm, (H) 1.0 mm.


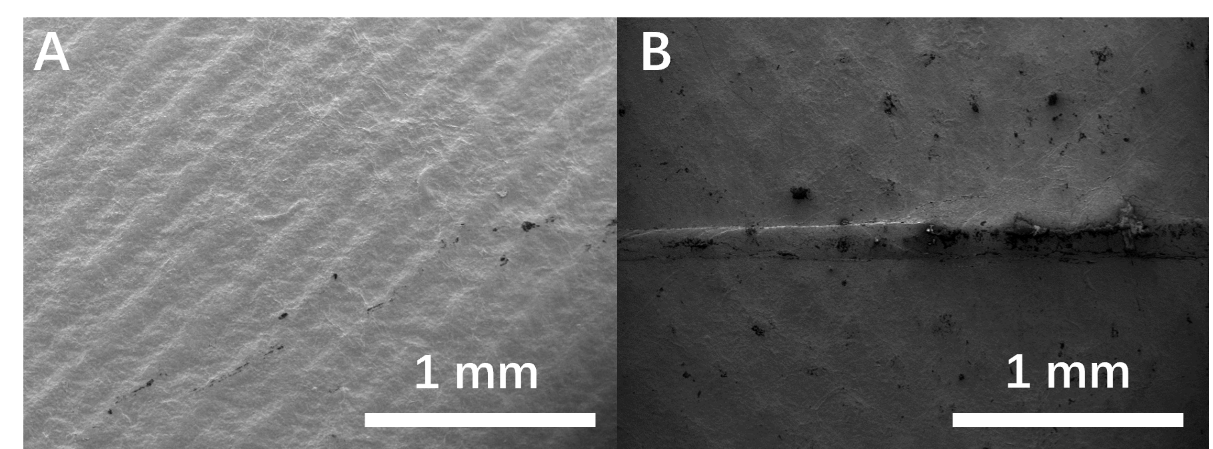


**Figure S9.** SEM images of gold-coated KBC substrates (A) before and (B) after repeated folding.


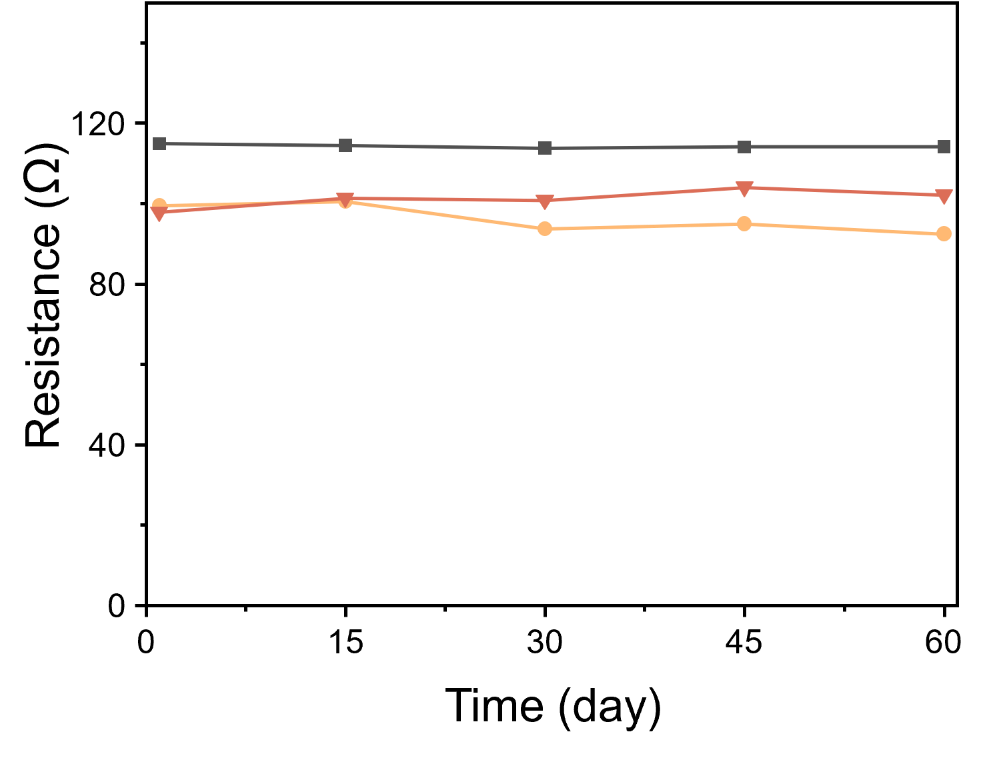


**Figure S10.** Resistance of gold-coated KBC substrates over two months at room humidity (80% RH).


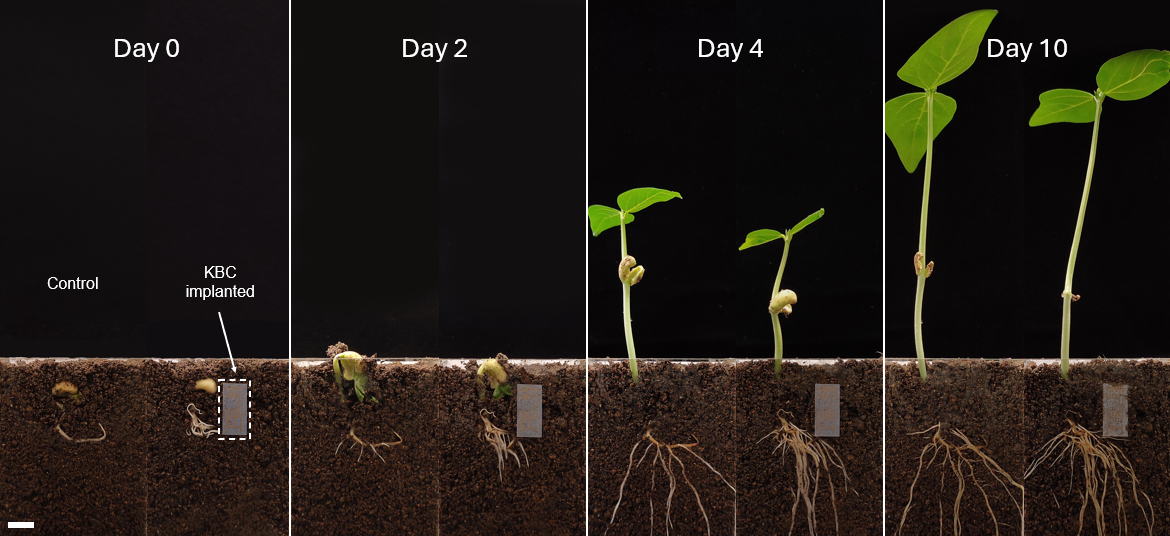

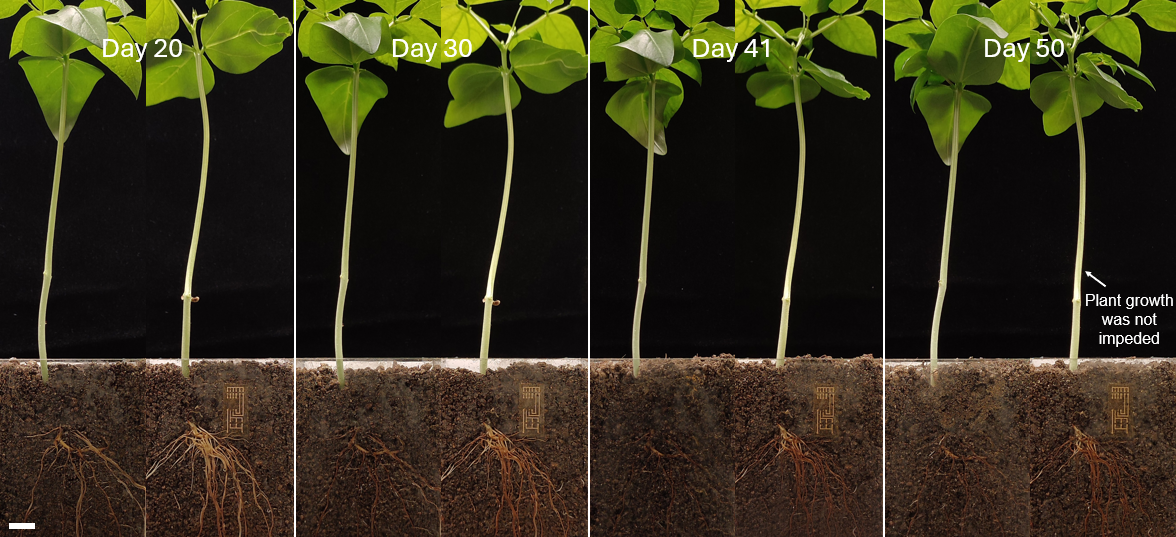


**Figure S11.** Time-lapse photography of the soil biodegradation process of KBC with a gold-sputtered circuit. The KBC was readily biodegraded and produced environmentally benign products that do not impede the growth of the plant. Visibility of the gold traces persisted until day 50, attributed to their adhesion to the glass container and non-biodegradability. Scale bar, 1cm.

**
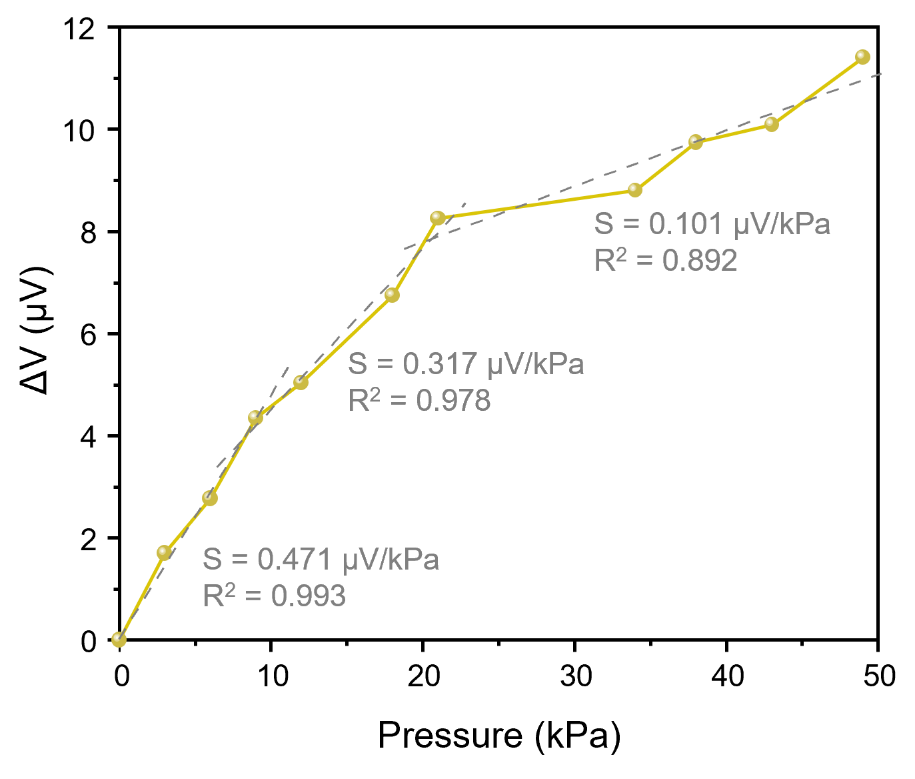
**

**Figure S12.** Sensitivity and linearity of the KBC-based pressure sensor.


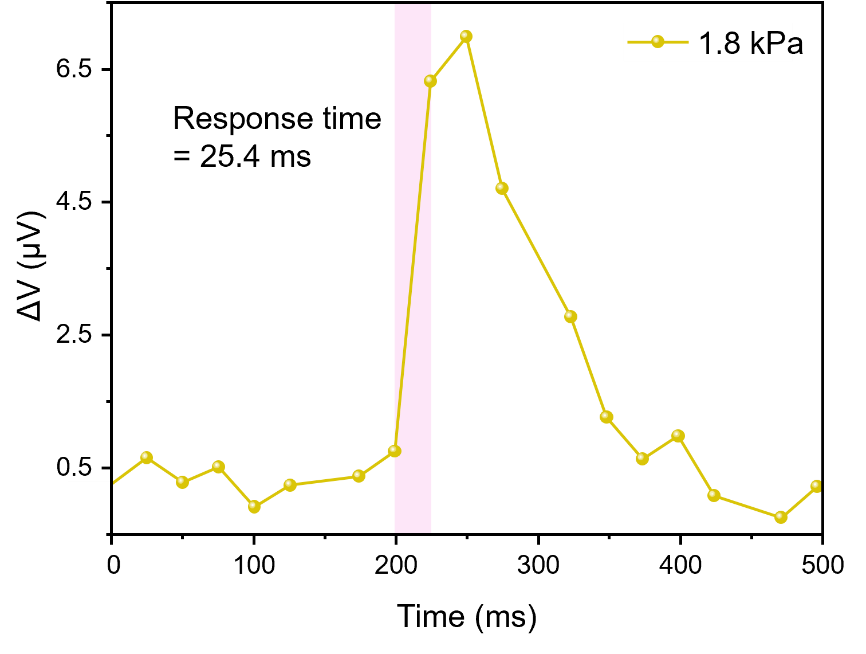


**Figure S13.** Response time of the KBC-based pressure sensor at 1.8 kPa loading.

1

7

10

16

21

26

31

0.0

0.5

1.0

1.5

2.0

2.5

Weight (g)

Time (day)

3

13

**Figure S14.** Weight changes of the KBC-based pressure sensor in soil.

**Table S1.** Comparison of the mechanical properties and degradation of KBC with other biodegradable substrates.

| **Substrate Material** | **Tensile Strength (MPa)** | **Young’s Modulus (GPa)** | | **Degradation** | **Ref.** |
| --- | --- | --- | --- | --- | --- |
| Paper | 60-100 | 6-8.5 | 2-6 weeks | | [1] |
| PLA | 40-85 | 2-3 | 6-24 months | | [2-4] |
| Silk composite | 15-40 | 0.4-1.5 | 5 min in hot water | | [5] |
| Starch | 6-12 | 0.10.9 | 30 days in soil | | [6] |
| Kombucha bacterial cellulose | 70-100 | 1-2 | 49 days | | **This work** |

**Table S2.** Designed and actually printed widths of gold circuits on the KBC substrate.

| Designed circuit width (mm) | Printed circuit width (mm) |
| --- | --- |
| 0.3 | 0.37 |
| 0.4 | 0.47 |
| 0.5 | 0.56 |
| 0.6 | 0.65 |
| 0.7 | 0.75 |
| 0.8 | 0.84 |
| 0.9 | 0.94 |
| 1.0 | 1.03 |

**References**

[1] Y. Mo, L. Yang, T. Zou, W. Hou, R. Liao, "Preparation of Composite Insulating Paper With Decreased Permittivity, Good Mechanical and Thermal Properties by Kevlar/Nano Cellulose Fibrils/Softwood Pulp Hybrid" IEEE Access 7 (2019): 104258-104268. <https://doi.org/https://doi.org/10.1109/access.2019.2930981>.

[2] G. Mattana, D. Briand, A. Marette, A. Vásquez Quintero, N. F. de Rooij, "Polylactic acid as a biodegradable material for all-solution-processed organic electronic devices" Organic Electronics 17 (2015): 77-86. <https://doi.org/https://doi.org/10.1016/j.orgel.2014.11.010>.

[3] R. A. Auras, B. Harte, S. Selke, R. Hernandez, "Mechanical, Physical, and Barrier Properties of Poly(Lactide) Films" Journal of Plastic Film & Sheeting 19(2) (2003): 123-135. <https://doi.org/https://doi.org/10.1177/8756087903039702>.

[4] M. Hussain, S. M. Khan, M. Shafiq, N. Abbas, "A review on PLA-based biodegradable materials for biomedical applications" Giant 18 (2024). <https://doi.org/https://doi.org/10.1016/j.giant.2024.100261>.

[5] M. Xu, H. Cai, Z. Liu, F. Chen, L. Chen, X. Chen, X. Cheng, F. Dai, Z. Li, "Breathable, Degradable Piezoresistive Skin Sensor Based on a Sandwich Structure for High‐Performance Pressure Detection" Advanced Electronic Materials 7(10) (2021). <https://doi.org/https://doi.org/10.1002/aelm.202100368>.

[6] M. Dong, A. Soul, Y. Li, E. Bilotti, H. Zhang, P. Cataldi, D. G. Papageorgiou, "Transient Starch‐Based Nanocomposites for Sustainable Electronics and Multifunctional Sensing" Advanced Functional Materials 35(1) (2024). <https://doi.org/https://doi.org/10.1002/adfm.202412138>.
